# Pharmacogenomics in pediatric oncology: Australian adolescent or young adult and caregiver perspectives

**DOI:** 10.1016/j.gimo.2025.103452

**Published:** 2025-09-03

**Authors:** Claire Moore, Emma F. Magavern, Marliese Alexander, Safeera Y. Hussainy, Tracey Danaher, Rishi S. Kotecha, Kyall Homberg, Marion K. Mateos, Sophie Jessop, Tayla Stenta, Dhrita Khatri, Elizabeth Williams, Roxanne Dyas, Julian Stolper, David A. Elliott, Rachel Conyers

**Affiliations:** 1 Cancer Therapies, Murdoch Children’s Research Institute, Parkville, Australia; 2 Department of Paediatrics, The University of Melbourne, Parkville, Australia; 3 Children’s Cancer Centre, The Royal Children’s Hospital, Parkville, Australia; 4 William Harvey Research Institute, Queen Mary University of London, London, United Kingdom; 5 Pharmacy Department, Peter MacCallum Cancer Centre, Parkville, Australia; 6 Sir Peter MacCallum Department of Oncology, The University of Melbourne, Parkville, Melbourne, Australia; 7 Department of General Practice, School of Public Health and Preventive Medicine, Faculty of Medicine, Nursing and Health Sciences, Monash University, Clayton, Victoria, Australia; 8 Monash University, Caufield East, Australia; 9 Children’s Cancer Foundation, Melbourne, Australia; 10 Department of Clinical Haematology, Oncology, Blood and Marrow Transplantation, Perth Children’s Hospital, Perth, Australia; 11 Leukaemia Translational Research Laboratory, WA Kids Cancer Centre, The Kids Research Institute Australia, University of Western Australia, Perth, Australia; 12 Curtin Medical School, Curtin University, Perth, Australia; 13 Kids Cancer Centre, Sydney Children’s Hospital Randwick, Sydney, Australia; 14 Children’s Cancer Institute, Lowy Cancer Research Centre, UNSW, Sydney, Australia; 15 School of Clinical Medicine, UNSW Medicine & Health, UNSW Sydney; 16 Michael Rice Department of Haematology and Oncology, Women's and Children's Hospital, Adelaide, Australia; 17 The Novo Nordisk Foundation Centre for Stem Cell Medicine, ReNEW, Melbourne Node, Parkville, Australia; 18 Australian Regenerative Medicine Institute, Monash University, Clayton, Victoria, Australia

**Keywords:** Adolescents, Caregivers, Oncology, Pharmacogenetics, Young adult

## Abstract

**Purpose:**

Preemptive pharmacogenomic (PGx) testing in pediatric oncology patients could reduce toxicity and improve efficacy of medications yet remains underutilized. Consumer identified implementation barriers have not been extensively explored nor included adolescent or young adult (AYA) patient perspectives. This study describes Australian pediatric oncology consumer perspectives on PGx testing, elucidating barriers to implementation.

**Methods:**

A theory-informed, quantitative survey was used to assess knowledge and attitudes toward PGx in 38 AYA patients and 66 caregivers. Additionally, AYAs were assessed for autonomy using a validated scale.

**Results:**

Participants viewed PGx testing positively, and 98% at least “somewhat agreed” that they would want to be tested/want their child to be tested. Concerns were identified in both AYA patient and caregiver groups, primarily the possibility of insurance discrimination and data security. Almost 90% of AYA patients expressed a desire for direct involvement in decisions regarding their PGx testing. However, the mean autonomy score (15.2) suggests that many felt they were not consistently given the opportunity to participate in decisions about their own lives.

**Conclusion:**

Despite positive attitudes toward PGx testing, pediatric oncology consumers still have some concerns that need to be addressed for successful implementation. Additionally, AYA patients should be included in PGx processes and implementation design.

## Introduction

Pharmacogenomics (PGx) is the use of individual genomic information to predict response to medications.^1^^,^^2^ Genetic factors influence both medication pharmacokinetics and pharmacodynamics, resulting in variability of efficacy and toxicity among individuals.^1^^,^^2^ Knowledge of PGx has been building over the last 3 decades, culminating in the formation of international consortia, such as the Clinical Pharmacogenetics Implementation Consortium (CPIC)^3^ and the Dutch Pharmacogenetic Working Group.^4^ These consortia have developed evidence-based guidelines for prescribing medications according to specific PGx genotype results. Although these guidelines could improve medication safety and efficacy, their widespread implementation into routine clinical care in pediatrics has been slow.^1^^,^^2^

In particular, PGx-guided prescribing has the potential to significantly benefit pediatric oncology patients. Children receiving cancer therapy are at increased risk of adverse effects because of the number and types of medications they are prescribed.^5^^,^^6^ It has been reported that 60% of pediatric oncology patients will experience an adverse drug reaction (ADR) during their cancer treatment, although this is likely underestimated.^5^ Using PGx to inform prescribing has reduced ADR occurrence in adults, and this utility could extend to children with cancer.^7^ However, few guidelines have routinely been incorporated into pediatric oncology clinical care. *TPMT/NUDT15*-guided prescribing for thiopurines in patients with acute lymphoblastic leukemia is the most commonly used guideline because of testing requirements mandated in clinical trials, such as those from the Children’s Oncology Group.^8^^,^^9^ Using this dosing approach has reduced the risk of life-threatening myelosuppression without compromising relapse.^10^^,^^11^ However, there are additional PGx prescribing guidelines from CPIC and Dutch Pharmacogenetic Working Group for multiple medications commonly used in pediatric oncology supportive care, including omeprazole, voriconazole, and ondansetron, which are not being widely utilized because of a number of complexities associated with PGx implementation.^12^, ^13^, ^14^

Barriers to the implementation of clinical PGx testing have been broadly described from a policy and infrastructure perspective.^2^ Insufficient PGx education, lack of access to consistent testing and reporting pathways, and the cost of PGx testing are identified as challenges.^1^^,^^2^ However, specific consumer-identified barriers to PGx testing must also be considered to ensure the success of implementation programs. More broadly, the importance of consumer involvement in genomic medicine is now recognized, with well-documented benefits.^15^^,^^16^

A 2023 systematic review of the knowledge, attitudes, and practice surrounding pharmacogenomics in pediatric oncology identified the paucity of studies seeking to identify PGx implementation barriers from a consumer perspective.^11^ The review included only 1 study with caregiver participants.^17^ This US-based study found that caregivers were concerned with PGx test cost, insurance discrimination, data sharing, and privacy.^17^ There were no patients themselves included in this study, despite the potential for Adolescent and Young Adult (AYA) patient involvement. Engagement with patients and caregivers regarding PGx testing is critical to bring the potential of personalized prescribing to the bedside.

This study aimed to identify barriers to PGx in pediatric oncology from an Australian AYA patient and caregiver perspective and to compare findings with those reported in the literature. It addresses the need to give AYA patients a voice in the mounting discourse around PGx implementation.

## Materials and Methods

### Study design

A cross-sectional, descriptive survey was designed and implemented to assess the knowledge and attitudes of pediatric oncology consumers with respect to PGx to identify consumer barriers to implementation. Separate surveys were designed for AYA patient participants and caregiver participants. This study received ethical approval from the Royal Children’s Hospital Human Research Committee (RCH/HREC/89089) and informed consent was obtained from consumers before commencing the surveys.

### Codesigning the survey

Survey design methodology was adapted from methods for designing survey tools described by Alexander et al.^18^ Key themes and attributes of consumer perceptions of pharmacogenomics in pediatric oncology were identified through a systematic review of the literature.^11^ These were combined with additional themes identified as salient by the authors through engagement with current literature, as well as those that emerged from discussions with a consumer engagement group (volunteer members from The Victorian Paediatric Cancer Consortium Patient and Family Advisory Committee). In this forum, pharmacogenomics in pediatric oncology was conceptually introduced, and committee members were invited to share their perspectives and attitudes. Findings from the systematic review were subsequently presented and committee members were given an additional opportunity to discuss.

Key themes and attributes were mapped to domains of the Theoretical Domains Framework.^19^ Survey questions were subsequently constructed to allow for identification of theory-based behavioral determinants influencing implementation. The survey draft was then subject to a peer review process, in which 2 collaborators from the Peter MacCallum Cancer Centre, 1 from Queen Mary University of London, and an Australian consumer representative with survey research experience were invited to provide feedback on survey question structure, language, and flow.

Survey questions were further refined through online consumer cognitive interview sessions. Each session was held with 2 consumer volunteers and 2 research members to assess the survey for face and content validity. Caregivers and AYA patients attended separate sessions. An initial session was conducted for each group; the survey was refined based on feedback, and then follow-up sessions with the same volunteers were held to ensure their feedback had been incorporated appropriately. Based on consumer feedback, the term “pharmacogenomics” was replaced with “DNA testing to guide medicine prescribing” throughout the survey.

**Figure 1:**
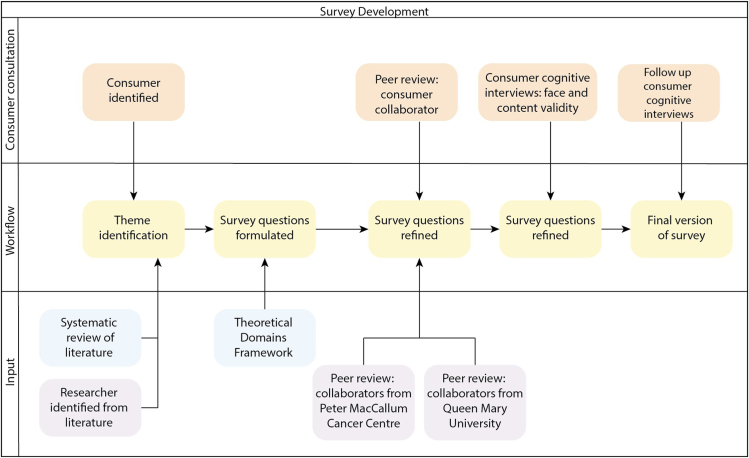
**Survey development methodology.** Figure is created with draw.io.^20^

The design process culminated in 2 separate anonymous and voluntary surveys designed to assess consumer knowledge and attitudes toward pharmacogenomics in pediatric oncology. One survey was designed for caregivers and the other for AYA patients. The process was completed over a 6-month period in 2023 and is summarized in Figure 1.^20^ There was a maximum of 24 content questions and 9 demographic questions. The first 2 questions were designed to assess baseline knowledge and attitudes toward PGx in pediatric oncology. This was followed by a publicly available, 3-minute video produced by the Garvan Institute of Medical Research (https://www.youtube.com/watch?v=Ar_Slk9M1SA) to broadly introduce PGx, using poor and rapid metabolizers of *CYP2D6* and codeine as an example and^21^ then a mixed-media (visual and text) explanation of how PGx can further be used for supportive care medication prescribing in pediatric oncology. Subsequently, a series of questions was asked (Figure 1^20^). The AYA survey contained an additional 6 questions from a validated scale measuring autonomy in adolescence from a control perspective (AAC), to determine whether autonomy was correlated with knowledge and attitudes^22^ (Supplemental Methods 1). Most questions contained options for response using a 7-point Likert scale (strongly disagree, disagree, somewhat disagree, neither agree nor disagree, somewhat agree, agree, and strongly agree). The survey can be accessed in full in the supplemental data (Supplemental Methods 2 and 3).

### Patient population

Inclusion criteria for caregivers were defined as having a child of any age who has had or is receiving treatment for cancer or an immune condition. Multiple caregivers of the same child could participate. Inclusion criteria for AYA patients were having had or currently undergoing treatment for cancer or an immune condition and aged 15 years or above (upper limit 39 years). The caregivers and AYA participants were not necessarily connected. The survey was only available in English. In both cases, enrolment in the Minimizing Adverse Drug Reactions and Verifying Economic Legitimacy-Pharmacogenomic Implementation in Children (MARVEL-PIC) study excluded participation. MARVEL-PIC is a broader Australian PGx implementation study currently underway, assessing the impact of preemptive pharmacogenomic testing on the incidence of ADRs in pediatric oncology patients.^6^ Patients who consent to MARVEL-PIC are provided extensive information on PGx screening and therefore not considered representative of the general patient population. Patients who had experience with TPMT/NUDT15 testing were not excluded.

### Survey dissemination and results

The survey was built using the REDCap database and incorporated branching logic. The survey was advertised on posters with QR codes at Australian pediatric oncology sites in Victoria, New South Wales, Western Australia, and South Australia in 2024. Demographics variables are reported by counts, percentages, means, and standard deviation as appropriate. Survey responses are reported as counts and percentages and analyzed separately for AYA patients and caregivers, as well as overall where possible.

## Results

### Participant details

There were 104 participants from 4 institutes in Australia. There were 38 AYA patients who began the survey, and 31 of these completed it (82%). There were 66 caregivers who began the survey, and 51 of these completed it (77%). Demographic details of the participants are included in Supplemental Table 1.

### Baseline assessment

**Figure 2:**
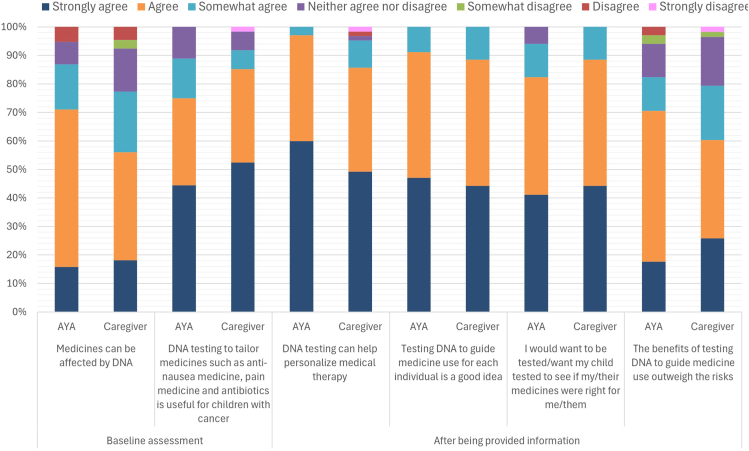
**Consumer knowledge and attitudes toward PGx in pediatric oncology.** AYA, adolescent or young adult; PGx, pharmacogenomics.

Without being provided with information on what PGx is, most participants at least “somewhat agreed” that medicines can be affected by DNA (87% of AYAs and 77% of caregivers) (Figure 2, Supplemental Table 2). Similarly, more than 90% of total participants at least “somewhat agreed” that PGx testing for antinausea medicine, pain medicine, and antibiotics is useful for children with cancer (86% of AYAs and 92% of caregivers). Approximately half of the participants in both groups “strongly agreed” with the statement (Figure 2).

### Participant knowledge and attitudes toward PGx in pediatric oncology

Participants were then asked to watch the introductory video by the Garvan Institute of Medical Research^21^ and given an example of how PGx can be used in supportive care prescribing for pediatric oncology patients. All AYA participants at least “somewhat agreed” that PGx testing can help personalize medical therapy by decreasing side effects of medications and improving the way they work, with 60% “strongly agreeing” (Figure 2, Supplemental Table 3). Among caregivers, 95% at least “somewhat agreed” and almost half “strongly agreed.” All participants thought that PGx testing for each individual is a good idea (at least “somewhat agreeing”). The majority of AYA participants expressed a desire to undergo PGx testing themselves, with 95% at least “somewhat agreeing” they would want to be tested, including. 41% that “strongly agreed.” Interestingly, all caregivers at least “somewhat agreed” that they would want their child to undergo PGx testing. Among these, 44% “strongly agreed.” Few participants reported substantial experience with PGx, with only 3% of AYAs and 6% of caregivers disclosing a health care professional had spoken to them “a lot” about PGx testing, and around half of both groups reported “not at all” (Supplemental Table 4). Only 12% of overall participants responded definitively that someone in their family had undergone PGx testing.

### Concerns around PGx testing in pediatric oncology

**Figure 3:**
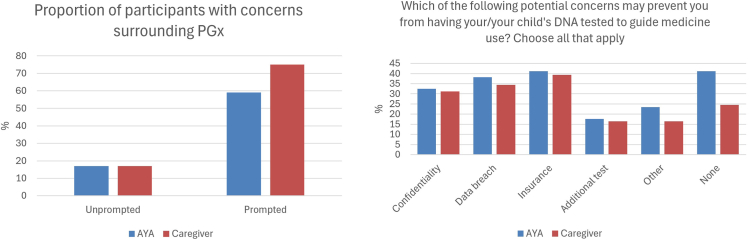
**Consumer concerns around PGx testing.** AYA, adolescent or young adult; PGx, pharmacogenomics.

Without outlining possible concerns with PGx testing, participants were asked whether they had any concerns. Interestingly, 17% of both groups reported having concerns surrounding PGx testing (Figure 3, Supplemental Table 5). Participants who answered affirmatively were asked to list their concerns. Responses included data sharing, data security, insurance discrimination, and education.

When participants were directly asked whether specific potential barriers (confidentiality, data breach, impact on health or life insurance, an additional test for the patient, or other long-term implications) may prevent them/their child from undergoing PGx testing, the proportion reporting at least 1 concern increased to 70% overall (AYA 59%, caregiver 75%). Prompting with these specific issues appeared to heighten awareness, leading to a 53% increase in the number of respondents identifying at least 1 potential concern. Insurance was the most noted concern with 41% of AYAs and 39% of caregivers reporting insurance implications may prevent them from undergoing PGx testing. Insurance was also ranked the most important concern in both groups.

Participants were then asked to consider whether the benefits of PGx testing outweighed the risks. Approximately 80% of both groups agreed with this statement.

### PGx education

**Figure 4:**
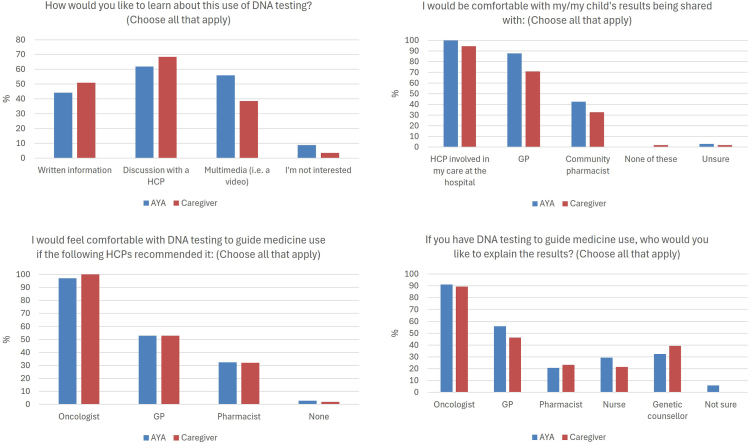
**Consumer responses to PGx education questions.** AYA, adolescent or young adult; GP, general practitioner; HCP, health care professional; PGx, pharmacogenomics.

Participants’ most preferred method of education about PGx was via a health care professional (HCP); AYAs 62% and caregivers 68% (Figure 4, Supplemental Table 6). The second preferred method was multimedia for the AYAs and written information for the caregivers. Interestingly, all AYAs and 95% of caregivers reported being comfortable with their PGx results shared with HCPs involved in their care within the hospital, but this number dropped significantly when considering a general practitioner (GP); 88% of AYAs and 71% of caregivers and further again for a community pharmacist; 42% of AYAs and 33% of caregivers A similar trend was observed when asked who participants would like to explain PGx results, with oncologist the preferred choice, followed by GP, genetic counselor, pharmacist, and nurse (Figure 4). In kind, participants reported being most comfortable with PGx testing if their oncologist recommended it, compared with their GP or pharmacist.

### AYA autonomy in PGx testing decisions

**Figure 5:**
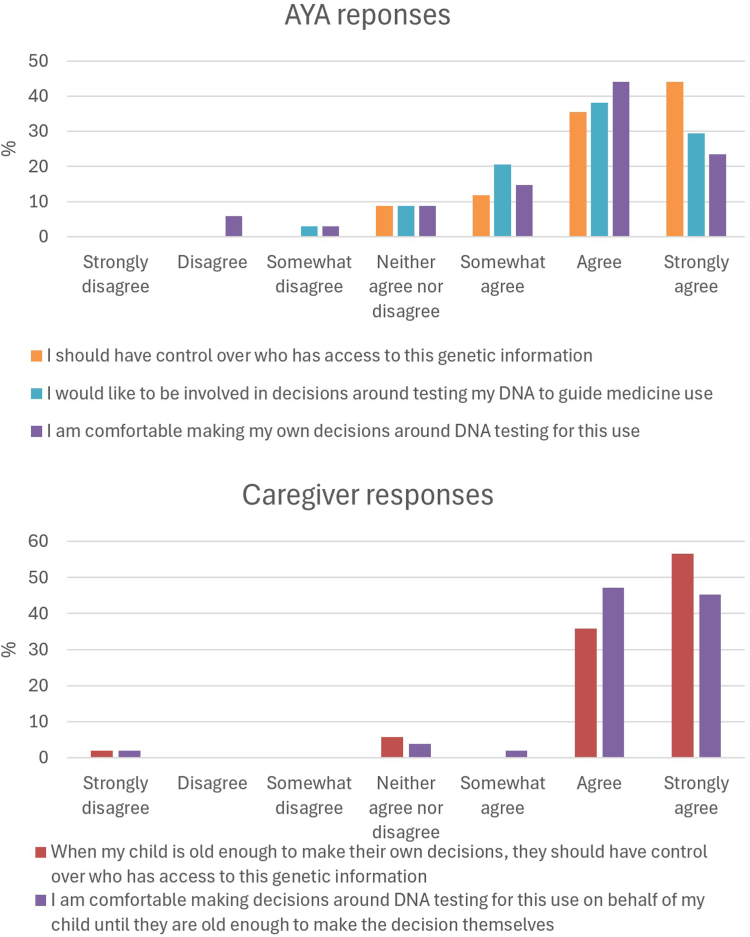
**Consumer responses to questions around autonomy and decision making in PGx testing.** AYA, adolescent or young adult; PGx, pharmacogenomics.

There were 91% of AYA participants that at least “somewhat agreed” that they should have control over who has access to their PGx data (Figure 5, Supplemental Table 7). Representative responses when asked why they felt this way included: “Big Pharma cannot be trusted,” “It is important to maintain control over personal information,” “It’s my personal information,” “It’s my body so my choice” (Supplemental Figure 1). Although most patients felt comfortable making PGx testing decisions on their own, there were differences in preferences and confidence. Two participants “disagreed,” 1 “somewhat disagreed,” and a further 3 “neither agreed nor disagreed.” Using the AAC scale to assess autonomy, participants scored between 9 and 22, mean 15.2 (*n* = 33, SD = 3.2) (Supplemental Table 8).

### Caregiver perspectives on decision making around PGx testing

The vast majority of caregivers (92%) either “agreed” or “strongly agreed” that their child should have control over who has access to their PGx information when they are old enough to make their own decisions (Figure 5, Supplemental Table 9). One caregiver “strongly disagreed,” sharing that their child has an intellectual disability. Although 94% of caregivers were comfortable making PGx testing decisions on behalf of their child until they can make decisions themselves, 1 caregiver did not, and 2 “neither agreed nor disagreed” with the statement.

## Discussion

Our AYA patient and caregiver survey participants expressed overwhelmingly positive attitudes toward pharmacogenomics in pediatric oncology, despite limited exposure to it, which is in keeping with the US study by Mowbray et al.^17^ It is not surprising that the benefits of preemptive PGx prescribing were apparent to the participants. In kind, positive attitudes toward genetic testing have been described in parents across a range of clinical settings, particularly in cases in which it was felt that there was a proven clinical benefit for their child.^23^ Ensuring patients are well informed regarding PGx testing and likely benefits will be key to their willingness to engage with the process.

Although the positive attitudes toward PGx are encouraging, 17% of both carers and AYAs reported concerns with aspects of PGx implementation (unprompted), and this number rose to 59% of AYAs and 75% of caregivers when prompted with potential concerns. The most common concerns identified in both groups were the possibility of insurance discrimination and data security. Interestingly, both of these were independently identified by the participants before being given a list of “potential concerns.” Insurance and data security were also found to be barriers in the study by Mowbray et al.^17^ Insurance discrimination is a well-documented consumer concern in other clinical genetic settings, as identified by persons at risk of Huntington’s disease and parents of children being screened for monogenetic or autosomal dominant disorders.^24^, ^25^, ^26^, ^27^ According to the *Disability Discrimination Act 1992* (Cth), in Australia, discrimination on the basis of genetic test results is prohibited, although life insurance, income protection, permanent disability, and travel insurance have previously been exempt where “justified.”^28^ In 2024, Australia announced a plan for legislating a total ban on the use of genetic test results in life insurance underwriting.^29^ This total ban would ameliorate this patient concern around genetic testing, although to date, legislative action has not been taken.

Data security was another significant concern of participants. Although not unique to pharmacogenomics, this consumer concern has been repeatedly raised in the context of pharmacogenomics.^30^^,^^31^ Qualitative research exploring the Australian public’s views on storing and sharing genomic data revealed many concerns.^32^ Overcoming this barrier will require adequate policies and infrastructure, ensuring appropriate safeguards for patients’ genomic data. The Australian Research Code dictates that genomic researchers follow proper practices for safety and security, and it is imperative we do so to gain public trust in genomic medicine.^33^

A point of difference in the perspectives of Australian and US caregivers regarding PGx testing in pediatric oncology was the emphasis placed by US parents on the cost of PGx testing.^17^ This was not self-identified as a concern by any of the participants in our study, who may have assumed testing would be Medicare funded, and may reflect differences in funding models between the US and Australian health care systems. However, survey participants were not directly asked whether test payment would affect their attitudes toward testing because of concerns around response bias. Only 3 PGx tests are currently funded by the Australian Government Medicare system, *TPMT* with thiopurine use, *HLA-∗57:01* with abacavir, and *DPYD* with 5-fluorouracil and capecitabine.^34^ Outside of these indications, patients pay for PGx tests privately as an out-of-pocket cost in primary care, although some centers will provide *NUDT15* genotyping alongside *TPMT* at no additional cost. Although a cytochrome P450 enzyme gene encoding panel costs patients just under $200 AUD, it is expected that costs will decrease once pharmacogenomics is more widely implemented and tests become rebated.^35^ However, with the increasing availability of next-generation sequencing, such as genome sequencing (GS) for oncology patients for clinical diagnostic purposes and to guide cancer treatment, an opportunity for repurposing these data for PGx analysis exists at no additional cost to the patients.

To improve the implementation of the authors’ existing pharmacogenomic studies,^6^ we wanted to understand the PGx education needs of respondents. It is clear that a multimodal approach to educating patients and their families will need to be used, taking into consideration the different educational needs and preferences for different audiences (ie, AYA patients compared with caregivers). The finding that the preferred method of PGx education be delivered by a HCP was unsurprising and aligns with findings from other studies describing HCPs as the primary source for patients seeking PGx information.^36^^,^^37^ Seeking education from a HCP is underpinned by trust and rapport.^36^^,^^37^ However, in an area of medicine that could be led by a variety of craft groups (eg, pharmacists, oncologists, and genetic counselors), it is less clear which HCP should take responsibility. Almost all the survey participants reported being comfortable with PGx test results being shared with their oncologist and would prefer their oncologist recommend PGx testing and deliver results. Involvement of a GP in these areas was second preference for the consumers, whereas the option of pharmacist involvement was significantly less supported. Although these findings may reveal who pediatric oncology consumers believe is best placed to guide PGx screening processes, the essence of the patient-oncologist relationship may skew perceptions. It has been suggested that the very nature of cancer and its treatment may force patients to trust their oncologists almost unconditionally.^38^ Additionally, research has shown that AYAs are highly connected to their oncologists, which has certainly been reflected in our results.^39^ Yet, pharmacists are well placed to play an integral role in the provision of clinical PGx services, given their expertise in medications and medication counseling. Pharmacists have demonstrated PGx leadership clinically and in research, through the inception of organizations such as the Pharmacogenomics Global Research Network and CPIC. In some centers, they are involved in initiating or ordering PGx tests, counseling patients, interpreting treatment recommendations, consulting with prescribers, and serving as an expert resource to patients and HCPs.^40^ Provided that pharmacists are adequately trained for these roles, they could be at the forefront of PGx medicine implementation. However, this would also require patient education around this role and close collaboration with the primary oncologist to build patient trust.

We addressed an existing research gap through gaining AYA patient perspectives on PGx implementation in pediatric oncology. The AYA phase of life can be complex even without cancer treatment, and the defining autonomy and independence that generally emerges at this time can be threatened by a diagnosis of cancer.^41^ Key developmental milestones may not be met in AYA patients with cancer because of the physical and psychosocial impacts of treatment and cancer itself, leading to reduced quality of life compared with the general population.^41^, ^42^, ^43^ Prior research has demonstrated the desire of AYA patients to exert control over their health care, expressing that, at the very least, they want to share decision making with their oncologist and caregivers.^39^^,^^44^ In our survey, most AYA patients reported that they want to be involved in decisions regarding PGx testing and are comfortable making PGx testing decisions independently, in keeping with their developmental need for autonomy. Disparity between desire for control in adolescence and legal capacity for medical decision making can be challenging for patients, families, and clinicians.^42^ When we measured autonomy from a control perspective in this cohort, using a validated tool^22^ to measure perceived possibilities for adolescents to influence their life, patients scored between 9 and 22 (from a maximum of 24), with a mean score of 15.2. This score is significantly lower than the mean scores observed in the validation of this tool (Female: 21.06, Male 21.47), when completed by 154 Swedish high school students aged 15 to 16.^22^ Although the AYA cohort in our study was small, which could skew the mean observed score, this difference could also be explained by the regression to caregiver reliance that many AYA patients report after a cancer diagnosis^44^ and highlights a critical opportunity for AYA patients to be involved in decisions around PGx testing. Including AYA patients in decision making has previously been shown to increase autonomy and even increase adherence to medical treatment.^45^^,^^46^ One study looking at perspectives of AYA patients with cancer on genomic research found that they want to be involved both at the time of consent and relaying of results, and they could recall and understand the results.^47^ We have an ethical duty to include AYA patients, where appropriate, in PGx processes that apply to them specifically, as well as in the design of implementation programs.

### Limitations

Given the exploratory design of this study and limited statistical power, findings should be interpreted as hypothesis generating rather than conclusive. The small numbers of both caregivers and AYA participants mean that their perspectives may not be representative of the broader pediatric oncology community. Additionally, the survey was only available in English, excluding non-English speakers who may provide valuable insights into barriers to PGx testing from the perspective of patients who are widely underrepresented in genomics.

Although pediatric oncology is a distinct population, aspects of the consumer perspectives on pharmacogenomics presented in this study may be relevant to additional underserved groups who stand to benefit from clinical genomics. Although applicability within other contexts needs confirmation, our findings reflect those of a 2017 systematic review of almost 4000 parents’ attitudes toward genetic testing in children across a range of genetics.^23^ This review also found that parents viewed genetic tests positively in cases in which there is perceived clinical benefit and perceived the primary disadvantage as potential employment or insurance discrimination.^23^

### Conclusion

Findings from this study reveal that our sample of AYA patients with cancer and caregivers from Australia have mostly positive attitudes toward PGx testing and would support implementation into clinical care. However, a significant proportion also identified potential barriers, including insurance discrimination and data security. These findings highlight the need for policy makers to address these concerns to strengthen implementation efforts. Similarities in the responses between groups regarding attitudes and concerns highlight the insight of AYA patients into PGx testing and their capacity to engage in shared decision making. AYAs want to be involved in PGx decision making and should play an active role in the design and implementation of these interventions intended for them.

## Data Availability

The data that support the findings of this study are available from the corresponding author (R.C.) upon request in situations in which participant consent has been given.

## ORCIDs

Claire Moore: http://orcid.org/0000-0001-7253-7913

Emma F. Magavern: http://orcid.org/0000-0003-0699-6411

Marliese Alexander: http://orcid.org/0000-0001-5782-7912

Safeera Y. Hussainy: http://orcid.org/0000-0003-1418-8078

Tracey Danaher: http://orcid.org/0000-0002-7248-0889

Rishi S. Kotecha: http://orcid.org/0000-0003-1836-4075

Kyall Homberg: http://orcid.org/0009-0000-8479-8416

Marion K. Mateos: http://orcid.org/0000-0002-2763-5853

Sophie Jessop: http://orcid.org/0000-0001-6403-0186

Tayla Stenta: http://orcid.org/0000-0003-4794-030X

Dhrita Khatri: http://orcid.org/0009-0004-3096-6022

Elizabeth Williams: http://orcid.org/0009-0003-7692-2837

Roxanne Dyas: http://orcid.org/0009-0005-1826-2257

Julian Stolper: http://orcid.org/0000-0001-7233-2686

David A. Elliott: http://orcid.org/0000-0003-1052-7407

Rachel Conyers: http://orcid.org/0000-0002-2344-1365

## Conflict of Interest

The authors declare no conflicts of interest.
