# Supplementary material for: Pharmacogenomics in pediatric oncology: Australian adolescent or young adult and caregiver perspectives
Source: Genet Med Open. 2025 Sep 3;3:103452. doi: 10.1016/j.gimo.2025.103452 (PMC12552976; doi:10.1016/j.gimo.2025.103452)
Supplement: Supplementary Material [file mmc1.pdf]

Supplementary material

Supplementary Table 1: **Demographics of participants.**

38 AYA patients began the survey and 31 completed it. 66 caregivers began the survey and 51 completed it.

	**AYA**	**Caregiver**	**Overall**
**Age:**	**(n=30)**	**(n=51)**	
Range	12-38	27-55	
Mean (SD)	17.7 (6.1)	51 (41.5)	
**Sex:**	**(n= 32)**	**(n=50)**	
Female	16 (50%)	41 (82%)	
Male	15 (47%)	9 (18%)	
Other	1 (3%)		
**Country of birth:**	**(n=32)**	**(n=51)**	
Australia	26	36	
Bangladesh	1	-	
India	1	1	
Malaysia	1	1	
South Africa	2	2	
U.S.A.	1	-	
China	-	1	
United Kingdom	-	2	
France	-	1	
Iran	-	2	
Iraq	-	1	
Japan	-	1	
Spain	-	1	
Vietnam	-	1	
U.S.S.R.	-	1	
**Language most often spoken at home:**	**(n=32)**	**(n=50)**	
Afrikaans	2	1	
Cantonese	1	-	
English	28	44	
Malayalam	1	-	
Englusy	-	1	
Farsi	-	1	
Punjabi	-	1	
Russian	-	1	
Vietnamese	-	1	
**Cultural background (could choose more than one):**	**(n=31)**	**(n=44)**	
Australian	6	13	

European<br>Asian<br>Aboriginal<br>South African<br>Indian<br>Arabic<br>Middle Eastern<br>None	15<br>4<br>2<br>2<br>2<br>-<br>-<br>-	15<br>7<br>4<br>1<br>-<br>1<br>2<br>1	
**Type of cancer/immune condition:**<br>Leukaemia/lymphoma<br>Solid tumour<br>CNS<br>Other	**(n=31)**<br><br>23<br>5<br>4<br>-	**(n=51)**<br><br>29<br>13<br>5<br>4	
**When was the condition treated:**<br>Current-within 6 months<br>6-12 months ago<br>>12 months<br>>3yrs	**(n=32)**<br><br>17<br>5<br>5<br>5	**(n=51)**<br><br>27<br>6<br>12<br>6	

Supplementary Table 2: **Baseline assessment.**

Medicines can be affected by DNA.							
	**Strongly disagree**	**Disagree**	**Somewhat disagree**	**Neither agree nor disagree**	**Somewhat agree**	**Agree**	**Strongly agree**
AYA (n=38)	0	2 (5.3%)	0	3 (7.9%)	6 (15.8%)	21 (55.3%)	6 (15.8%)
Caregiver (n=66)	0	3 (4.6%)	2 (3%)	10 (15.2%)	14 (21.2%)	25 (37.9%)	12 (18.2%)
Total (n=104)	0	5 (4.8%)	2 (1.9%)	13 (12.5%)	20 (19.2%)	46 (44.2%)	18 (17.3%)
DNA testing to tailor medicines such as anti-nausea medicine, pain medicine and antibiotics is useful for children with cancer.							
	**Strongly disagree**	**Disagree**	**Somewhat disagree**	**Neither agree nor disagree**	**Somewhat agree**	**Agree**	**Strongly agree**
AYA (n=36)	0	0	0	4 (11.1%)	5 (13.9%)	11 (30.6%)	16 (44.4%)

Caregiver (n=61)	1 (1.6%)	0	0	4 (6.6%)	4 (6.6%)	20 (32.8%)	32 (52.5%)
Total (n=97)	1 (1%)	0	0	8 (8.2%)	9 (9.3%)	31 (32%)	48 (49.5%)

Supplementary Table 3: **Participant knowledge of and attitudes towards PGx in paediatric oncology.**

DNA testing can help personalize medical therapy (by decreasing side effects of medications and improving the way they work).							
	**Strongly disagree**	**Disagree**	**Somewhat disagree**	**Neither agree nor disagree**	**Somewhat agree**	**Agree**	**Strongly agree**
AYA (n=35)	0	0	0	0	1 (2.9%)	13 (37.1%)	21 (60%)
Caregiver (n=63)	1 (1.6%)	1 (1.6%)	0	1 (1.6%)	6 (9.5%)	23 (36.5%)	31 (49.2%)
Total (n=98)	1 (1%)	1 (1%)	0	1 (1%)	7 (7.1%)	36 (36.7%)	52 (53%)
Testing DNA to guide medicine use for each individual is a good idea.							
	**Strongly disagree**	**Disagree**	**Somewhat disagree**	**Neither agree nor disagree**	**Somewhat agree**	**Agree**	**Strongly agree**
AYA (n=34)	0	0	0	0	3 (8.8%)	15 (44.1%)	16 (47.1%)
Caregiver (n=61)	0	0	0	0	7 (11.5%)	27 (44.3%)	27 (44.3%)
Total (n=95)	0	0	0	0	10 (10.5%)	42 (44.2%)	43 (45.3%)
I would want to be tested/want my child tested to see if my/their medicines were right for me/them.							
	**Strongly disagree**	**Disagree**	**Somewhat disagree**	**Neither agree nor disagree**	**Somewhat agree**	**Agree**	**Strongly agree**
AYA (n=34)	0	0	0	2 (5.9%)	4 (11.8%)	14 (41.2%)	14 (41.2%)
Caregiver (n=61)	0	0	0	0	7 (11.5%)	27 (44.3%)	27 (44.3%)

Total (n=95)	0	0	0	2 (2.1%)	11 (11.6%)	41 (43.2%)	41 (43.2%)

Supplementary Table 4: **Experience with PGx testing.**

A healthcare professional has spoken to me about testing DNA to guide medicine use:				
	**A lot**	**A little**	**Not at all**	**Unsure**
AYA (n=35)	1 (2.9%)	10 (28.6%)	16 (45.7%)	8 (22.9%)
Caregiver (n=63)	4 (6.3%)	24 (38.1%)	32 (50.8%)	3 (4.8%)
Total (n=98)	5 (5.1%)	34 (34.7%)	48 (49%)	11 (11.2%)

Someone in my family has had DNA tested to guide their medicine use.			
	**Yes**	**No**	**Unsure**
AYA (n=34)	4 (11.8%)	19 (55.9%)	11 (32.4%)
Caregiver (n=55)	7 (12.7%)	38 (69.1%)	10 (18.2%)
Total (n=89)	11 (12.4%)	57 (64%)	21 (23.6%)

Supplementary Table 5: **Concerns around PGx testing in paediatric oncology.**

Do you have any concerns about the use of DNA testing to guide medicine use?			
	**No**	**Yes**	**If yes, what? (number of responses)**
AYA (n=34)	28 (82.4%)	6 (17.6%)	Discrimination of some sort (i.e. insurance) (2)<br>Data security (2)<br>Data misuse (i.e. third party) (3)
Caregiver (n=58)	48 (82.8%)	10 (17.2%)	Additional tests (1)<br>Data misuse (2)<br>Unanswered questions around process (2)<br>Data security (1)<br>Confidentiality (1)<br>Discrimination (insurance) (1)<br>Unsure of efficacy (1)
Total (n=92)	76 (82.6%)	16 (17.4%)	As above

Which of the following potential concerns may prevent you from having your/your child's DNA tested to guide medicine use (choose all that apply).						
	**Long term implications**	**Confidentiality**	**Data breach**	**Insurance**	**Additional test**	**None**
AYA (n=34)	8 (23.5%)	11 (32.4%)	13 (38.2%)	14 (41.2%)	6 (17.6%)	14 (41.2%)
Caregiver (n=61)	10 (16.4%)	19 (31.1%)	21 (34.4%)	24 (39.3%)	10 (16.4%)	15 (24.5%)
Total (n=95)	18 (18.9%)	30 (31.6%)	34 (35.8%)	38 (40%)	16 (16.8%)	29 (30.5%)

The benefits of testing DNA to guide medicine use outweigh the risks.							
	**Strongly disagree**	**Disagree**	**Somewhat disagree**	**Neither agree nor disagree**	**Somewhat agree**	**Agree**	**Strongly agree**
AYA (n=34)	0	1 (2.9%)	1 (2.9%)	4 (11.8%)	4 (11.8%)	18 (52.9%)	6 (17.6%)
Caregiver (n=57)	1 (1.8%)	0	1 (1.8%)	10 (17.5%)	11 (19.3%)	20 (35.1%)	15 (26.3%)
Total (n=91)	1 (1.1%)	1 (1.1%)	2 (2.2%)	14 (15.4%)	15 (16.5%)	38 (41.8%)	21 (23.1%)

Supplementary Table 6: **PGx education.**

How would you like to learn about this use of DNA testing? (Choose all that apply)				
	**Written information**	**HCP discussion**	**Multimedia (ie a video)**	**I'm not interested**
AYA (n=34)	15 (44.1%)	21 (61.8%)	19 (55.9%)	3 (8.8%)
Caregiver (n=57)	29 (50.9%)	39 (68.4%)	22 (38.6%)	2 (3.5%)
Total (n=91)	44 (48.4%)	60 (65.9%)	41 (45.1%)	5 (5.5%)

I would be comfortable with my/my child's results being shared with (choose all that apply):					
	**HCP involved in my care at the hospital**	**GP**	**Community pharmacist**	**None of these**	**Unsure**
AYA (n=33)	33 (100%)	29 (87.9%)	14 (42.4%)	0	1 (3%)

Caregiver (n=55)	52 (94.5%)	39 (70.9%)	18 (32.7%)	1 (1.8%)	1 (1.8%)
Total (n=88)	85 (96.6%)	68 (77.3%)	32 (36.4%)	1 (1.1%)	2 (2.3%)

I would feel comfortable with DNA testing to guide medicine use if the following HCPs recommended it (choose all that apply):

	**Oncologist**	**GP**	**Pharmacist**	**None**
AYA (n=34)	33 (97.1%)	18 (52.9%)	11 (32.4%)	1 (2.9%)
Caregiver (n=53)	52 (100%)	27 (52.8%)	16 (32.1%)	1 (1.9%)
Total (n=87)	85 (97.8%)	45 (51.7%)	27 (31%)	2 (2.3%)

If you have DNA testing to guide medicine use, who would you like to explain the results? (Choose all that apply)

	**Oncologist**	**GP**	**Pharmacist**	**Nurse**	**Genetic counsellor**	**Not sure**
AYA (n=34)	31 (91.2%)	19 (55.9%)	7 (20.6%)	10 (29.4%)	11 (32.4%)	2 (5.9%)
Caregiver (n=56)	50 (89.3%)	26 (46.4%)	13 (23.2%)	12 (21.4%)	22 (39.3%)	0
Total (n=90)	81 (90%)	45 (50%)	20 (22.2%)	22 (24.4%)	33 (36.7%)	2 (2.2%)

Supplementary Table 7: **AYA autonomy in PGx testing decisions.**

I should have control over who has access to this genetic information.

	**Strongly disagree**	**Disagree**	**Somewhat disagree**	**Neither agree nor disagree**	**Somewhat agree**	**Agree**	**Strongly agree**
AYA (n=34)	0	0	0	3 (8.8%)	4 (11.8%)	12 (35.5%)	15 (44.1%)

I would like to be involved in decisions around testing my DNA to guide medicines use.

	**Strongly disagree**	**Disagree**	**Somewhat disagree**	**Neither agree nor disagree**	**Somewhat agree**	**Agree**	**Strongly agree**
AYA (n=34)	0	0	1 (2.9%)	3 (8.8%)	7 (20.6%)	13 (38.2%)	10 (29.4%)

I am comfortable making my own decisions around DNA testing for this use.

	**Strongly disagree**	**Disagree**	**Somewhat disagree**	**Neither agree**	**Somewhat agree**	**Agree**	**Strongly agree**

				**nor disagree**			
AYA (n=34)	0	2 (5.9%)	1 (2.9%)	3 (8.8%)	5 (14.7%)	15 (44.1%)	8 (23.5%)

Supplementary Methods 1: **Autonomy from a control perspective (validated scale):**

(1) *Do you find that adults don't listen to you? (reversed)* (2) *Do you take part in important decisions concerning your life?* (3) *Do you find yourself treated unjustly because of your age? (reversed)* (4) *Do you feel that adults take you seriously?* (5) *Do you wish you could have a greater influence on your daily life?* (6) *Do you think your life would be better if more decisions were up to you?* Responses were given on a five-point Likert-type scale to each question with alternatives from 0 ('never') to 4 ('always'). A high value indicated high autonomy.

<u>Note: correspondence with author of the scale led to adjustments in the way the above assessment was scored. See below (email dated 28/11/23):</u>

Dear Claire

You are welcome to use the AAC scale, and the wording in the article is correct. However, item 1, 3 and 5 should be reversed, a typo missed to indicate this for item 5.

Good luck with your research

Mårten (Eriksson)

Supplementary Table 8: **AYA participant responses to AAC questions (n=33)**

Qn. 1	Qn. 2	Qn. 3	Qn. 4	Qn. 5	Qn. 6	AAC:
Rarely	Always	Never	Often	Often	Sometimes	17
Sometimes	Often	Sometimes	Sometimes	Often	Sometimes	12
Sometimes	Always	Rarely	Often	Never	Never	16
Often	Sometimes	Sometimes	Rarely	Often	Sometimes	9
Sometimes	Always	Sometimes	Often	Sometimes	Sometimes	15
Sometimes	Often	Sometimes	Always	Often	Sometimes	14
Often	Often	Often	Sometimes	Always	Always	11
Sometimes	Sometimes	Sometimes	Sometimes	Sometimes	Sometimes	12
Rarely	Always	Rarely	Often	Sometimes	Rarely	16
Sometimes	Sometimes	Sometimes	Often	Always	Often	12
Never	Always	Rarely	Always	Rarely	Always	22
Never	Always	Never	Always	Never	Never	20
Rarely	Often	Never	Often	Always	Always	17
Sometimes	Always	Sometimes	Sometimes	Often	Sometimes	13

Often	Always	Rarely	Often	Sometimes	Sometimes	15
Sometimes	Always	Rarely	Sometimes	Always	Sometimes	13
Rarely	Often	Never	Often	Rarely	Often	20
Rarely	Always	Rarely	Often	Rarely	Never	16
Sometimes	Often	Rarely	Often	Sometimes	Sometimes	15
Rarely	Often	Rarely	Often	Rarely	Sometimes	17
Sometimes	Often	Never	Often	Rarely	Sometimes	17
Sometimes	Often	Rarely	Sometimes	Sometimes	Sometimes	14
Rarely	Always	Never	Often	Rarely	Rarely	18
Rarely	Always	Never	Always	Never	Never	19
Sometimes	Sometimes	Rarely	Often	Sometimes	Sometimes	14
Never	Always	Never	Always	Rarely	Never	19
Rarely	Always	Never	Always	Rarely	Never	18
Sometimes	Always	Often	Sometimes	Sometimes	Sometimes	13
Sometimes	Sometimes	Sometimes	Sometimes	Often	Sometimes	11
Sometimes	Sometimes	Rarely	Sometimes	Often	Sometimes	12

Never	Always	Never	Always	Rarely	Rarely	20
Rarely	Often	Rarely	Rarely	Rarely	Rarely	14
Sometimes	Sometimes	Sometimes	Sometimes	Sometimes	Sometimes	12

Supplementary Table 9: **Caregiver perspectives on decision making around PGx testing.**

When my child is old enough to make their own decisions, they should have control over who has access to this genetic information.							
	**Strongly disagree**	**Disagree**	**Somewhat disagree**	**Neither agree nor disagree**	**Somewhat agree**	**Agree**	**Strongly agree**
Caregiver (n=53)	1 (1.9%)	0	0	3 (5.7%)	0	19 (35.8%)	30 (56.6%)
I am comfortable making decisions around DNA testing for this use on behalf of my child until they are old enough to make the decision themselves.							
	**Strongly disagree**	**Disagree**	**Somewhat disagree**	**Neither agree nor disagree**	**Somewhat agree**	**Agree**	**Strongly agree**
Caregiver (n=53)	1 (1.9%)	0	0	2 (3.8%)	1 (1.9%)	25 (47.2%)	24 (45.3%)

Supplementary Figure 1: **AYA patient perspectives on wanting control over who can access their PGx data.**

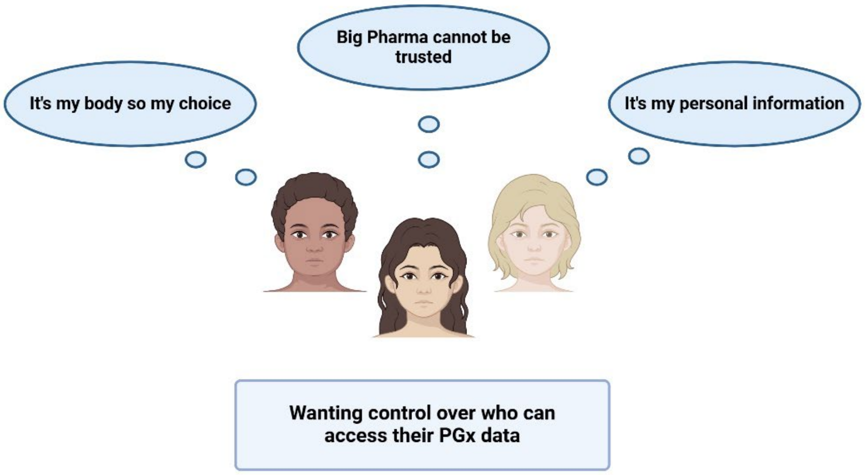

Supplementary Methods 2: Caregiver PGx survey.

Caregiver attitudes towards DNA testing to guide medicine use in children with cancer

Thank you for your interest in completing this survey. Please note this survey is only available in English.What is this survey about?This survey will help us to understand your views as a caregiver on DNA testing to guide medicine use in children with cancer (or who are immunosuppressed). This testing is not widely available in Australia. By completing this survey, you will help us understand how we can best provide access to this testing to improve how medicines are prescribed. What do you need to do?Complete the survey, which includes watching a short introductory video on DNA testing to guide medicine use. You will be asked questions on what you know about this topic and what you think about it. Your responses will help us understand how we can improve access to DNA testing to guide medicine use. The entire survey will take 5-10 minutes. Please complete within 24 hours of beginning the survey.Who is conducting the research?This study is being conducted by researchers at the Murdoch Children's Research Institute (MCRI). Contact: pharmaco.genomics@mcri.edu.au About the surveys:To be eligible to participate, 1. You should be a caregiver of a child who has received a diagnosis of cancer or immunosuppression (current or recent) AND
2. Your child should NOT be enrolled in the MARVEL-PIC study.These surveys are anonymous and voluntary. By providing survey answers, you are confirming you are eligible to participate and you consent to having your data stored securely on MCRI servers and de-identified answers published for research purposes. If you no longer wish to participate, please disregard this survey.Prize draw.People who complete the survey can enter a prize draw for one of three $50 gift cards. This is optional. In order to enter this draw, please ensure you complete all questions in the survey within 24 hours. On completion you will be provided with a separate link to enter your email address. This email address will be used solely for the purpose of notifying you if you have won a gift card (unless we obtain your permission otherwise).

Time survey started ______________________________

First we would like to ask some general questions:

Medicines can be affected by DNA.

◯ Strongly disagree
◯ Disagree
◯ Somewhat disagree
◯ Neither agree nor disagree
◯ Somewhat agree
◯ Agree
◯ Strongly agree

DNA testing to tailor medicines such as anti-nausea medicine, pain medicine and antibiotics is useful for children with cancer.

◯ Strongly disagree
◯ Disagree
◯ Somewhat disagree
◯ Neither agree nor disagree
◯ Somewhat agree
◯ Agree
◯ Strongly agree

Please watch this short introductory video, which talks about how DNA impacts medicines.

The testing in this video is different from the routine genetic testing of cancer cells to choose cancer targeting medicines and is also different from genetic testing to diagnose or predict disease. The testing in this video is only talking about parts of your DNA from when you are born that can impact medicines commonly used to treat: nausea and vomiting, depression, heartburn, and infections. Testing can be done from blood or spit or hair and should not cause any pain or distress.

From this point in the survey, we will call this "DNA testing to guide medicine use".

If the video does not show below, it can be accessed at:
https://www.youtube.com/watch?app=desktop&v=GprwJChewDE

Now that you have watched the video, please look at the infographic below for an example of how DNA testing can guide medicine use in children with cancer.

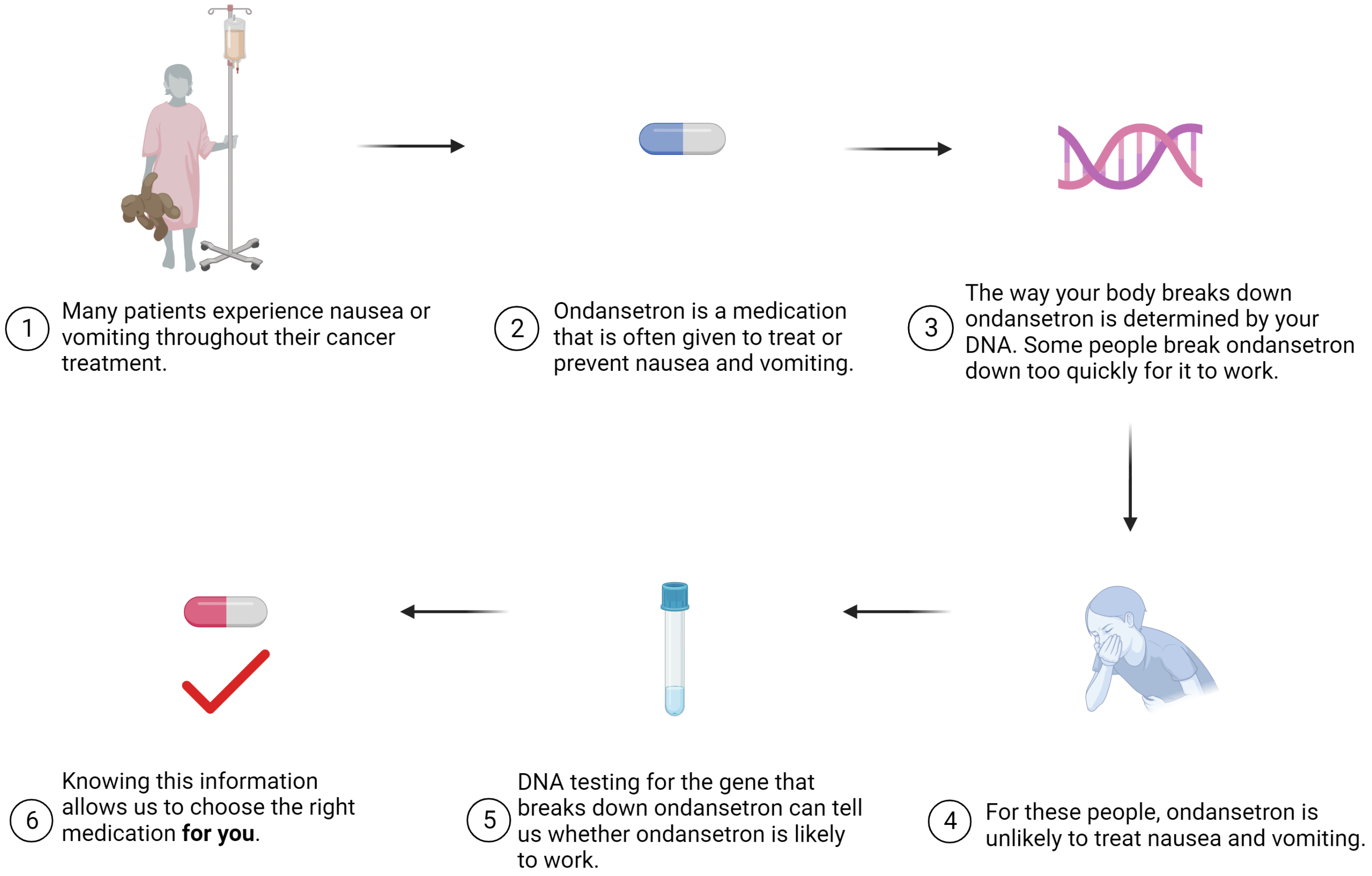

After watching the video and looking at the above infographic, please answer the following questions.

DNA testing can help personalize medical therapy (by decreasing side effects of medications and improving the way they work).

◯ Strongly disagree
◯ Disagree
◯ Somewhat disagree
◯ Neither agree nor disagree
◯ Somewhat agree
◯ Agree
◯ Strongly agree

A health care professional has spoken to me about testing DNA to guide medicine use.

◯ A lot
◯ A little
◯ Not at all
◯ Unsure

For the purpose of the questions below, we would like to discuss only the use of DNA to guide medicine use as you have seen in the video and infographic.

This use of DNA will not be able to tell you about genetic risk of disease, only about response to medicines.

Testing DNA to guide medicine use for each individual is a good idea.

◯ Strongly disagree
◯ Disagree
◯ Somewhat disagree
◯ Neither agree nor disagree
◯ Somewhat agree
◯ Agree
◯ Strongly agree

I would want my child tested to see if their medicines were right for them.

◯ Strongly disagree
◯ Disagree
◯ Somewhat disagree
◯ Neither agree nor disagree
◯ Somewhat agree
◯ Agree
◯ Strongly agree

We would like to understand what concerns you may have around testing DNA to guide medicine use. This is important to understand potential barriers and safeguards that could be put in place.

Do you have any concerns about the use of DNA testing to guide medicine use?

○ Yes ○ No

What are your concerns about the use of DNA testing to guide medicine use?

Which of the following potential concerns may prevent you from having your child's DNA tested to guide medicine use (choose all that apply).

☐ Long-term implications of my child having DNA testing to personalize medications
☐ Confidentiality of my child's DNA results when used within the hospital (for example in the electronic medical record)
☐ A data breach
☐ Impact on health or life insurance
☐ If an additional blood test was required
☐ None
☐ Other

If you answered "Other" in the previous question, please tell us what that concern is.

Calculate rank

____

From the concerns you selected in the last question, please rank in order of importance to you, listing your biggest concern as number 1. For example, if you selected three concerns, please rank them as 1,2 and 3.

(There won't be a ranking table if you had no concerns in the previous question)

	1	2	3	4	5	6
Long-term implications of my child having DNA testing to guide medicine use	◯	◯	◯	◯	◯	◯
Confidentiality of my child's DNA results	◯	◯	◯	◯	◯	◯
A data breach	◯	◯	◯	◯	◯	◯
Impact on health or life insurance	◯	◯	◯	◯	◯	◯
If an additional blood test was required	◯	◯	◯	◯	◯	◯
Other (as identified in the previous question)	◯	◯	◯	◯	◯	◯

The benefits of testing DNA to guide medicine use outweigh the risks.

◯ Strongly disagree
◯ Disagree
◯ Somewhat disagree
◯ Neither agree nor disagree
◯ Somewhat agree
◯ Agree
◯ Strongly agree

Now, we would like to know about your experience with DNA testing to guide medicine use.

This refers to testing DNA to guide medicines generally rather than testing cancer DNA to personalize anticancer treatment.

Someone in my family has had DNA tested to guide their medicine use.

◯ Yes
◯ No
◯ Unsure

Someone explained those results and what they meant.

◯ Strongly disagree
◯ Disagree
◯ Somewhat disagree
◯ Neither agree nor disagree
◯ Somewhat agree
◯ Agree
◯ Strongly agree

Do you know which medication the DNA testing was used for?

____

Now, we would like to explore communication and education on this topic.

How would you like to learn about this use of DNA testing? (choose all that apply)

☐ Written information
☐ Discussion with a healthcare professional
☐ Multimedia (eg a short video)
☐ I'm not interested
☐ Other

If you answered "Other" in the previous question, please tell us how you would like to learn about this topic.

____

If my child had DNA testing to guide medicine use, I would be comfortable with my child's results being shared with: (choose all that apply)

☐ Health professionals involved in my child's care within the hospital
☐ My child's General Practitioner (GP)
☐ Community pharmacist
☐ None of these
☐ Unsure

Now, we would like to ask some additional questions around DNA testing to guide medicine use.

This use of DNA will not be able to tell you about genetic risk of disease, only about response to medicines.

When my child is old enough to make their own decisions, they should have control over who has access to this genetic information.

◯ Strongly disagree
◯ Disagree
◯ Somewhat disagree
◯ Neither agree nor disagree
◯ Somewhat agree
◯ Agree
◯ Strongly agree

Please tell us why you have answered this way (optional).

I am comfortable making decisions around DNA testing for this use on behalf of my child until they are old enough to make the decision themselves.

◯ Strongly disagree
◯ Disagree
◯ Somewhat disagree
◯ Neither agree nor disagree
◯ Somewhat agree
◯ Agree
◯ Strongly agree

I would feel comfortable with DNA testing to guide medicine use for my child if the following healthcare professionals recommended it (choose all that apply):

☐ Oncologist
☐ GP
☐ Pharmacist
☐ None

If your child has DNA testing to guide medicine use, who would you like to explain the results? (choose all that apply)

☐ Oncologist
☐ GP
☐ Pharmacist
☐ Nurse
☐ Genetic counsellor
☐ Not sure

Is there anything else you would like to tell us about in relation to DNA testing to guide medicine use?

Now, we would like to know some information about you as the caregiver

What is your age?

____

What is your gender?

◯ Female ◯ Male ◯ Other

What is your postcode?

____

In which country were you born?

____

Which language do you most often speak at home?

____

What is your cultural background? (For example Australian Aboriginal, South-East Asian, Anglo-European)

____

What type of cancer/immune condition does your child have (or has your child had)?

____

When has your child last received treatment for this condition?

◯ Within the last 6 months (or currently having treatment) ◯ 6-12 months ago ◯ More than 12 months ago ◯ More than 3 years ago

How old is your child now?

____

You have reached the end of the official survey questions.

Time survey completed

____

Patient attitudes towards DNA testing to guide medicine use in children with cancer (Adolescents or Young Adults)

Thank you for your interest in completing this survey. Please note this survey is only available in English.

What is this survey about?

This survey will help us to understand your views on DNA testing to guide medicine use in children with cancer (or who are immunosuppressed). This testing is not widely available in Australia. By completing this survey, you will help us understand how we can best provide access to this testing to improve how medicines are prescribed.

What do you need to do?

Complete the survey, which includes watching a short introductory video on DNA testing to guide medicine use. You will be asked questions on what you know about this topic and what you think about it. You will also be asked questions about opportunities to make your own decisions. Your responses will help us understand how we can improve access to DNA testing to guide medicine use. The entire survey will take 5-10 minutes. Please complete within 24 hours of beginning the survey.

Who is conducting the research?

This study is being conducted by researchers at the Murdoch Children's Research Institute (MCRI). Contact: pharmaco.genomics@mcri.edu.au

About the surveys:

To be eligible to participate,

1. You should be 15 years of age (or above) AND

2. You should have a diagnosis of cancer or immunosuppression (current or recent) AND

3. You should NOT be enrolled in the MARVEL-PIC study.

These surveys are anonymous and voluntary. By providing survey answers, you are confirming you are eligible to participate and you consent to having your data stored securely on MCRI servers and de-identified answers published for research purposes. If you no longer wish to participate, please disregard this survey.

Prize draw.

People who complete the survey can enter a prize draw for one of three $50 gift cards. This is optional. In order to enter this draw, please ensure you complete all questions in the survey within 24 hours. On completion you will be provided with a separate link to enter your email address. This email address will be used solely for the purpose of notifying you if you have won a gift card (unless we obtain your permission otherwise).

Time survey started ______________________

First we would like to ask some general questions:

Medicines can be affected by DNA.

◯ Strongly disagree
◯ Disagree
◯ Somewhat disagree
◯ Neither agree nor disagree
◯ Somewhat agree
◯ Agree
◯ Strongly agree

DNA testing to tailor medicines such as anti-nausea medicine, pain medicine and antibiotics is useful for children with cancer.

◯ Strongly disagree
◯ Disagree
◯ Somewhat disagree
◯ Neither agree nor disagree
◯ Somewhat agree
◯ Agree
◯ Strongly agree

Please watch this short introductory video, which talks about how DNA impacts medicines.

The testing in this video is different from the routine genetic testing of cancer cells to choose cancer targeting medicines and is also different from genetic testing to diagnose or predict disease. The testing in this video is only talking about parts of your DNA from when you are born that can impact medicines commonly used to treat: nausea and vomiting, depression, heartburn, and infections. Testing can be done from blood or spit or hair and should not cause any pain or distress.

From this point in the survey, we will call this "DNA testing to guide medicine use".

If the video does not show below, it can be accessed at:
https://www.youtube.com/watch?app=desktop&v=GprwJChewDE

Now that you have watched the video, please look at the infographic below for an example of how DNA testing can guide medicine use in children with cancer.

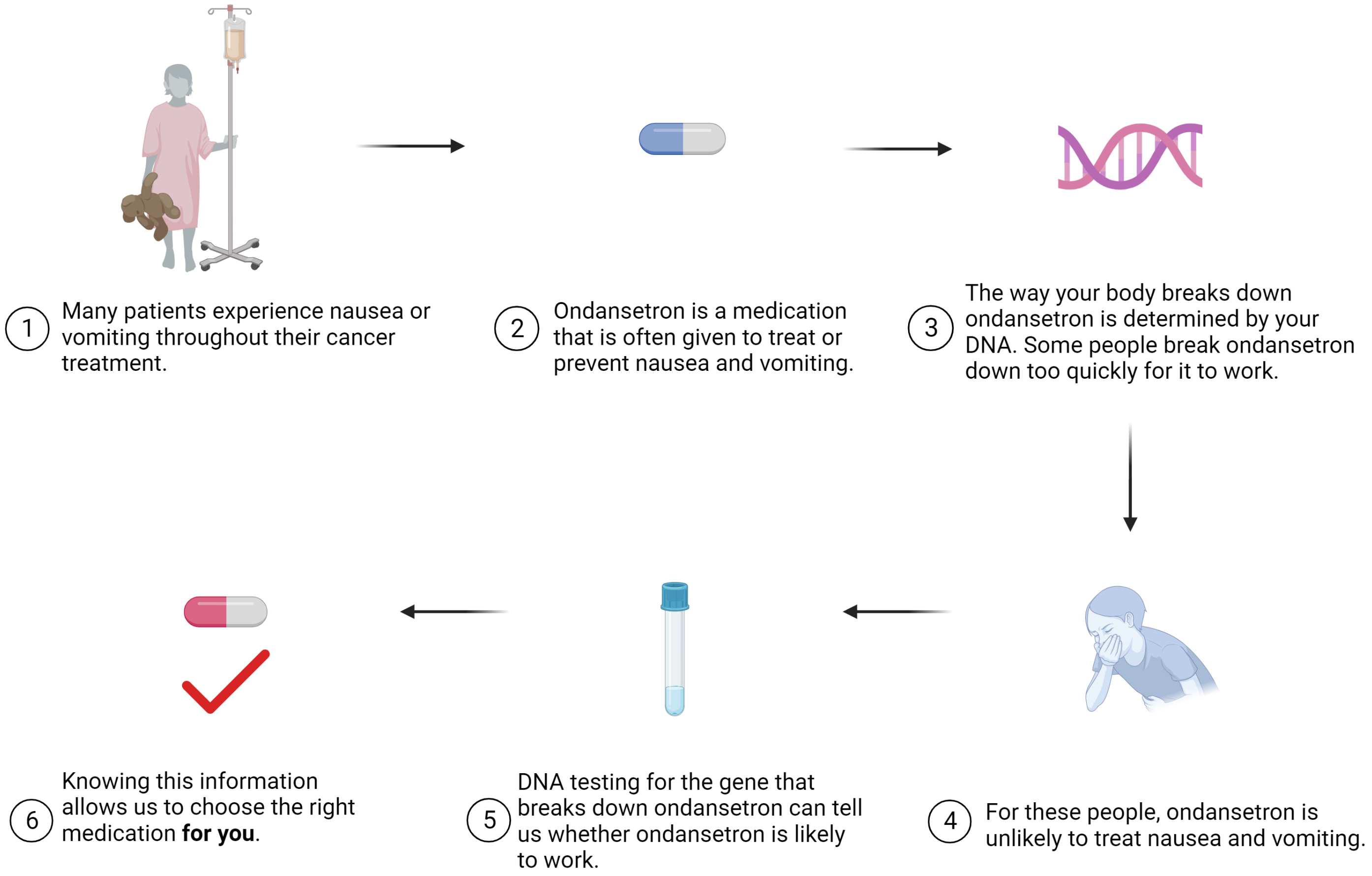

After watching the video and looking at the above infographic, please answer the following questions.

DNA testing can help personalize medical therapy (by decreasing side effects of medications and improving the way they work).

◯ Strongly disagree
◯ Disagree
◯ Somewhat disagree
◯ Neither agree nor disagree
◯ Somewhat agree
◯ Agree
◯ Strongly agree

A health care professional has spoken to me about testing DNA to guide medicine use.

◯ A lot
◯ A little
◯ Not at all
◯ Unsure

For the purpose of the questions below, we would like to discuss only the use of DNA to guide medicine use as you have seen in the video and infographic.

This use of DNA will not be able to tell you about genetic risk of disease, only about response to medicines.

Testing DNA to guide medicine use for each individual is a good idea.

◯ Strongly disagree
◯ Disagree
◯ Somewhat disagree
◯ Neither agree nor disagree
◯ Somewhat agree
◯ Agree
◯ Strongly agree

I would want to be tested to see if my medicines were right for me.

◯ Strongly disagree
◯ Disagree
◯ Somewhat disagree
◯ Neither agree nor disagree
◯ Somewhat agree
◯ Agree
◯ Strongly agree

We would like to understand what concerns you may have around testing DNA to guide medicine use. This is important to understand potential barriers and safeguards that could be put in place.

Do you have any concerns about the use of DNA testing to guide medicine use?

◯ Yes ◯ No

What are your concerns about the use of DNA testing to guide medicine use?

Which of the following potential concerns may prevent you from having your DNA tested to guide medicine use (choose all that apply).

- ☐ Long-term implications of having DNA testing to personalize medications
- ☐ Confidentiality of my DNA results when used within the hospital (for example in the electronic medical record)
- ☐ A data breach
- ☐ Impact on health or life insurance
- ☐ If an additional blood test was required
- ☐ None
- ☐ Other

If you answered "Other" in the previous question, please tell us what that concern is.

Calculate rank ______________________________

From the concerns you selected in the last question, please rank in order of importance to you, listing your biggest concern as number 1. For example, if you selected three concerns, please rank them as 1,2 and 3.

(There won't be a ranking table if you had no concerns in the previous question)

	1	2	3	4	5	6
Long-term implications of having DNA testing to guide medicine use	◯	◯	◯	◯	◯	◯
Confidentiality of my DNA results	◯	◯	◯	◯	◯	◯
A data breach	◯	◯	◯	◯	◯	◯
Impact on health or life insurance	◯	◯	◯	◯	◯	◯
If an additional blood test was required	◯	◯	◯	◯	◯	◯
Other (as identified in the previous question)	◯	◯	◯	◯	◯	◯

The benefits of testing DNA to guide medicine use outweigh the risks.

- ◯ Strongly disagree
- ◯ Disagree
- ◯ Somewhat disagree
- ◯ Neither agree nor disagree
- ◯ Somewhat agree
- ◯ Agree
- ◯ Strongly agree

Now, we would like to know about your experience with DNA testing to guide medicine use.

This refers to testing DNA to guide medicines generally rather than testing cancer DNA to personalize anticancer treatment.

Someone in my family has had DNA tested to guide their medicine use.

◯ Yes
◯ No
◯ Unsure

Someone explained those results and what they meant.

◯ Strongly disagree
◯ Disagree
◯ Somewhat disagree
◯ Neither agree nor disagree
◯ Somewhat agree
◯ Agree
◯ Strongly agree

Do you know which medication the DNA testing was used for?

____

Now, we would like to explore communication and education on this topic.

How would you like to learn about this use of DNA testing? (choose all that apply)

☐ Written information
☐ Discussion with a healthcare professional
☐ Multimedia (eg a short video)
☐ I'm not interested
☐ Other

If you answered "Other" in the previous question, please tell us how you would like to learn about this topic.

____

If I had DNA testing to guide medicine use, I would be comfortable with my results being shared with: (choose all that apply)

☐ Health professionals involved in my care within the hospital
☐ My General Practitioner (GP)
☐ Community pharmacist
☐ None of these
☐ Unsure

Now, we would like to ask some additional questions around DNA testing to guide medicine use.

This use of DNA will not be able to tell you about genetic risk of disease, only about response to medicines.

I should have control over who has access to this genetic information.

◯ Strongly disagree
◯ Disagree
◯ Somewhat disagree
◯ Neither agree nor disagree
◯ Somewhat agree
◯ Agree
◯ Strongly agree

Please tell us why you have answered this way (optional).

I would like to be involved in decisions around testing my DNA to guide medicines use.

◯ Strongly disagree
◯ Disagree
◯ Somewhat disagree
◯ Neither agree nor disagree
◯ Somewhat agree
◯ Agree
◯ Strongly agree

I am comfortable making my own decisions around DNA testing for this use.

◯ Strongly disagree
◯ Disagree
◯ Somewhat disagree
◯ Neither agree nor disagree
◯ Somewhat agree
◯ Agree
◯ Strongly agree

I would feel comfortable with DNA testing to guide medicine use if the following healthcare professionals recommended it (choose all that apply):

☐ Oncologist
☐ GP
☐ Pharmacist
☐ None

If you have DNA testing to guide medicine use, who would you like to explain the results? (choose all that apply)

☐ Oncologist
☐ GP
☐ Pharmacist
☐ Nurse
☐ Genetic counsellor
☐ Not sure

Is there anything else you would like to tell us about in relation to DNA testing to guide medicine use?

Now, we would like to get your thoughts on whether your age affects opportunities to make your own decisions.

This information will help us to suggest ways to empower adolescents and young adults in the setting of DNA testing to guide medicine use.

Do you find that adults don't listen to you?

◯ Never
◯ Rarely
◯ Sometimes
◯ Often
◯ Always

Do you take part in important decisions concerning your life?

◯ Never
◯ Rarely
◯ Sometimes
◯ Often
◯ Always

Do you find yourself treated unjustly because of your age?

◯ Never
◯ Rarely
◯ Sometimes
◯ Often
◯ Always

Do you feel that adults take you seriously?

◯ Never
◯ Rarely
◯ Sometimes
◯ Often
◯ Always

Do you wish you could have a greater influence on your daily life?

◯ Never
◯ Rarely
◯ Sometimes
◯ Often
◯ Always

Do you think your life would be better if more decisions were up to you?

◯ Never
◯ Rarely
◯ Sometimes
◯ Often
◯ Always

Now, we would like to know some information about you

What is your age?

____

What is your gender?

◯ Female ◯ Male ◯ Non-binary ◯ Other

Please specify

____

What is your postcode?

____

In which country were you born?

____

Which language do you most often speak at home?

____

What is your cultural background? (For example Australian Aboriginal, South-East Asian, Anglo-European)

____

What type of cancer/immune condition do you have (or have you had)?

____

When have you last received treatment for this condition?

◯ Within the last 6 months (or currently having treatment) ◯ 6-12 months ago ◯ More than 12 months ago ◯ More than 3 years ago

You have reached the end of the official survey questions.

Time survey completed

____
